# Gastric Perforation With Hepatic Involvement due to an Ingested Wooden Toothpick: A Case Report

**DOI:** 10.7759/cureus.85180

**Published:** 2025-06-01

**Authors:** Amador H Falconi Santiago, Michelle Cruz Méndez, Jorge Noceda Crispin, Emilio Mondragón Rosas, José Emiliano González Flores

**Affiliations:** 1 Division of Surgery, Department of Metabolic/Bariatric Surgery and Advanced Laparoscopy, Hospital Angeles Universidad, Mexico City, MEX; 2 Metabolic and Bariatric Surgery, Topilejo General Hospital, Mexico City, MEX; 3 Medical Education and Simulation, School of Medicine and Health Sciences, Tecnológico de Monterrey, Mexico City Campus, Mexico City, MEX; 4 Medicine, School of Medicine and Health Sciences, Tecnológico de Monterrey, Mexico City Campus, Mexico City, MEX

**Keywords:** foreign body migration, gastric perforation, hepatic involvement, hepatic penetration, toothpick ingestion

## Abstract

Toothpick ingestion is a rare but dangerous clinical event due to the risk of gastrointestinal perforation and migration into adjacent organs. Diagnosis is often delayed because patients rarely recall ingestion, and wooden toothpicks are radiolucent. We present a case of a 52-year-old man with epigastric pain and no clear etiology. CT imaging showed free air near the gastric body but did not reveal the foreign body. Exploratory laparotomy identified a wooden toothpick perforating the anterior gastric wall and segment II of the liver. Surgical repair was performed successfully, and the patient had a full recovery without complications. This case highlights the importance of clinical suspicion in patients with unexplained abdominal pain, particularly those with risk factors like alcohol use. Prompt surgical intervention is essential when imaging is inconclusive. Awareness of such rare presentations can help reduce diagnostic delays and improve patient outcomes.

## Introduction

Foreign body ingestion is a relatively frequent cause of emergency department visits, with most cases (60%-90%) resolving spontaneously and without complications [1]. However, ingestion of wooden toothpicks, though rare, is particularly dangerous due to their sharp, rigid structure. These objects can result in gastrointestinal perforation, bleeding, abscesses, or even death if not promptly managed [2]. In some cases, they may migrate into adjacent organs, leading to severe and potentially life-threatening complications. Common injury sites include the stomach, duodenum, cecum, sigmoid colon, jejunum, and colon, while the splenic flexure is rarely involved [3,4]. Diagnosis remains challenging, as most patients do not recall swallowing the object, delaying recognition and appropriate intervention.

We report the case of a 52-year-old man with silent toothpick ingestion leading to gastric perforation and hepatic involvement. The foreign body was identified intraoperatively and removed through exploratory laparotomy. The patient made a full recovery, highlighting the importance of clinical suspicion and timely surgical management in such atypical scenarios.

## Case presentation

The patient was a 52-year-old man who presented with acute abdominal pain. Symptoms began two days prior after waking up with abdominal pain in the epigastric and mesogastric regions radiating to the back. Nausea and vomiting occurred twice, prompting him to seek emergency care. He was admitted for observation and hospitalized for further evaluation. The patient denied any known allergies, chronic conditions, or prior surgical history. He was unaware of any history of blood transfusions. He reported a right radius fracture 15 years ago, which did not require surgical intervention. He had a history of chronic alcohol use spanning 20 years, during which he consumed both distilled and fermented alcoholic beverages every two days until reaching a state of intoxication. He denied tobacco use and reported occasional marijuana use.

The patient was afebrile, with mild pallor of the mucous membranes and skin. His head and neck examination revealed no abnormalities. Cardiopulmonary examination showed no apparent compromise. The abdomen was soft, slightly distended, and tender to deep palpation in the epigastric and mesogastric regions, with pain more pronounced in the epigastrium. Rebound tenderness was present, with mild voluntary guarding only on deep palpation. Bowel sounds were present and normoactive. Murphy’s sign was equivocal, while McBurney’s, Rovsing’s, and Blumberg’s signs were negative. Extremities exhibited adequate strength, sensitivity, and palpable pulses in all limbs.

**Figure 1:**
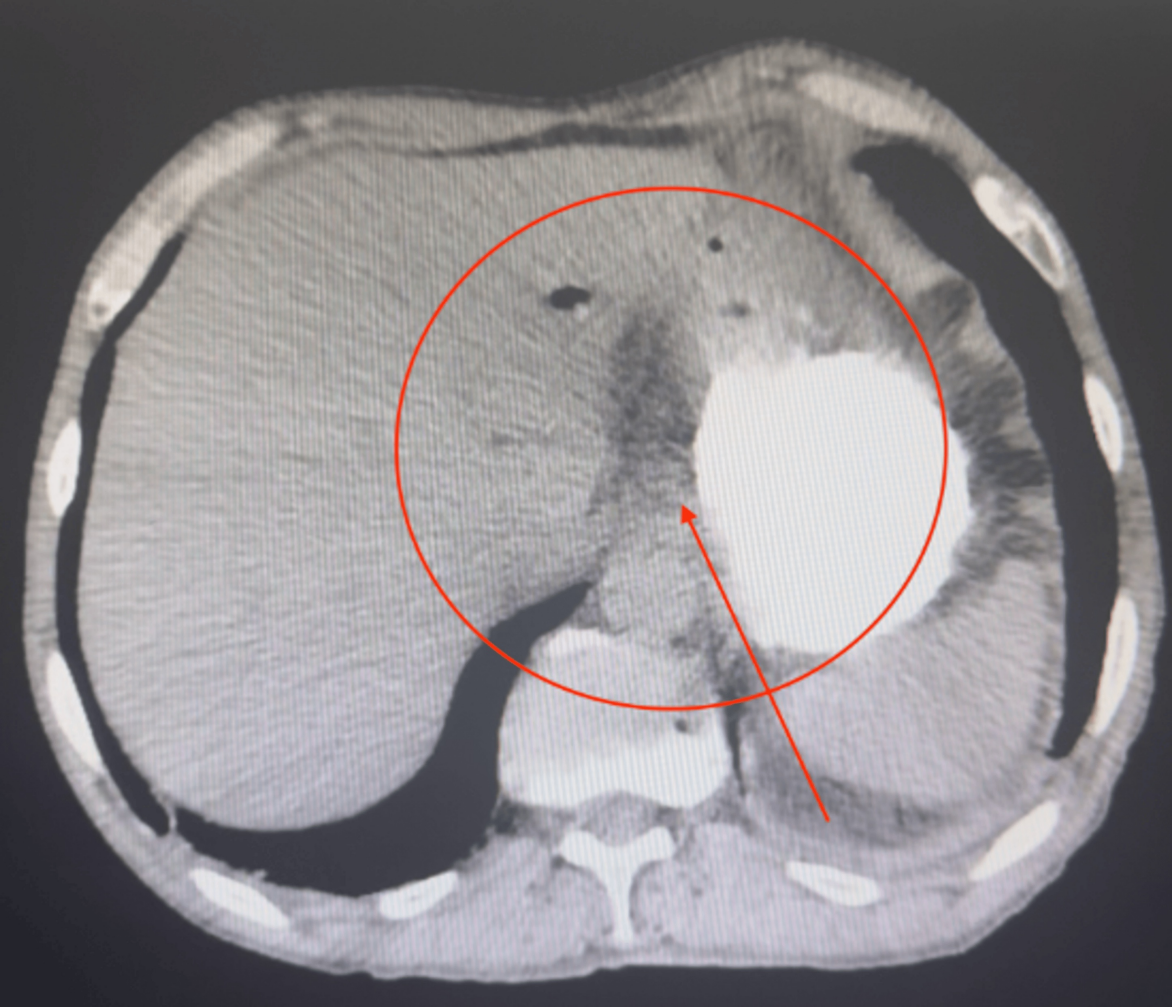
Axial CT scan of the abdomen with oral contrast showing free intraperitoneal air in the subhepatic space (highlighted by the red circle and arrow), adjacent to the anterior wall of the gastric body. Although the ingested wooden toothpick is not visible due to its radiolucency, this finding raised suspicion of a contained gastrointestinal perforation, leading to exploratory laparotomy.

During hospitalization, an abdominopelvic CT scan with oral contrast (Figure 1), and a full panel of laboratory tests were performed (Table 1), revealing the following: adequate passage of the contrast medium without evidence of leakage at any gastrointestinal level and possible free air were observed in the region of the gastric body within the subhepatic space, along with suspected pneumobilia. No other abnormalities were noted.

**Table 1:** Laboratory results of the patient compared with normal reference ranges for adult males WBC: white blood cells; Hb: hemoglobin; Hct: hematocrit; BUN: blood urea nitrogen; AST: aspartate aminotransferase; ALT: alanine aminotransferase; GGT: gamma-glutamyl transferase; ALP: alkaline phosphatase; LDH: lactate dehydrogenase; PT: prothrombin time Abnormal values may indicate underlying pathological processes and are essential for guiding clinical decision-making.

Lab test	Patient	Reference range (male)
WBC (×10⁹/L)	15.3	4.5–11
Neutrophils (%)	76.0	40–70
Hemoglobin (g/dL)	17.8	13.5–17.5
Hematocrit (%)	44.2	41–53
Platelets (×10⁹/L)	135.0	150–400
Glucose (mg/dL)	104.0	70–99
Creatinine (mg/dL)	1.2	0.74–1.35
BUN (mg/dL)	12.0	7–20
Urea (mg/dL)	24.0	15–40
Sodium (mmol/L)	141.4	135–145
Potassium (mmol/L)	3.4	3.5–5.1
Chloride (mmol/L)	105.0	98–107
Total bilirubin (mg/dL)	1.9	0.1–1.2
Direct bilirubin (mg/dL)	0.41	0–0.3
Indirect bilirubin (mg/dL)	1.49	0.2–0.9
AST (U/L)	37.0	10–40
ALT (U/L)	46.0	7–56
GGT (U/L)	39.0	8–61
ALP (U/L)	176.0	45–115
LDH (U/L)	189.0	140–280
PT (sec)	15	11–13.5
Albumin (g/dL)	2.95	3.4–5.4
Globulin (g/dL)	3.2	2–3.5
Amylase (U/L)	68.3	30–110
Lipase (U/L)	23.2	0–160

**Figure 2:**
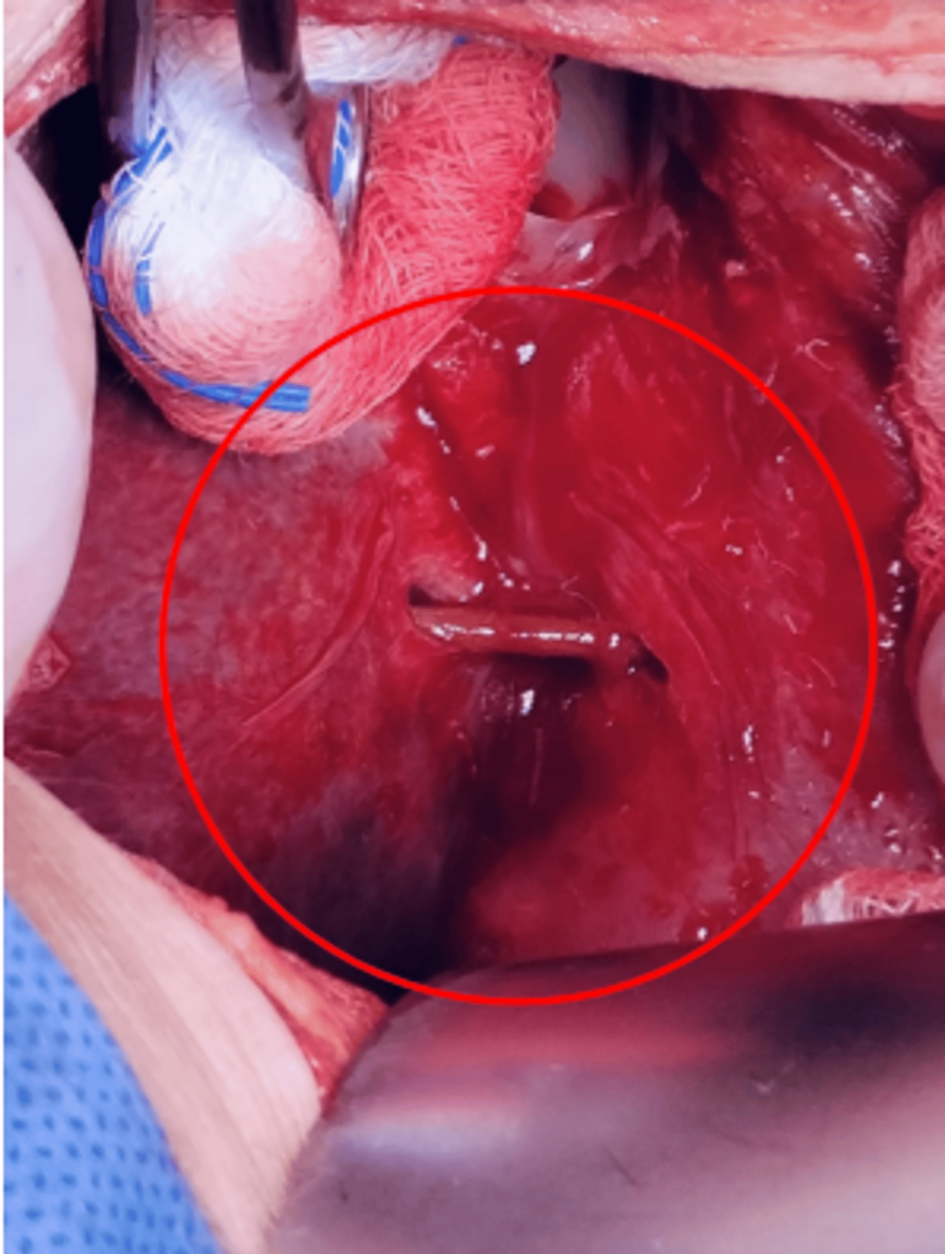
Intraoperative image showing a wooden foreign body (toothpick) (red circle) perforating the anterior wall of the stomach (right) at the junction of the body and antrum, along the lesser curvature. The object penetrates segment II of the liver (left) without evidence of active bleeding or gastrointestinal fluid leakage. The surrounding tissues exhibit signs of inflammation. This finding was managed with primary gastric repair in two layers and an omental patch.

**Figure 3:**
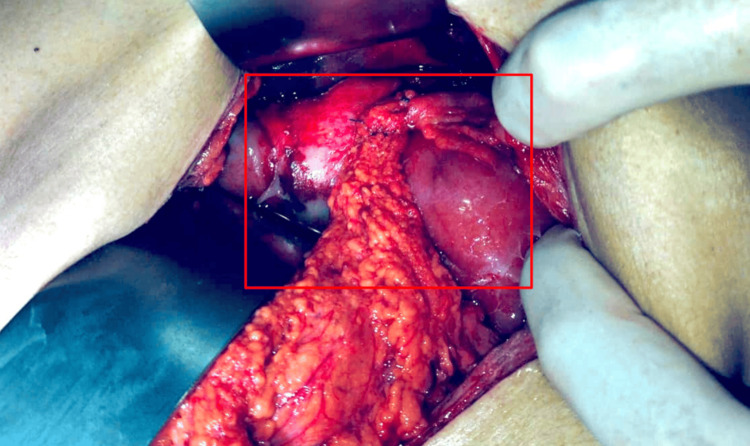
Intraoperative view showing anatomical sites affected by a penetrating wooden foreign body. The red box outlines the gastric perforation site at the anterior wall near the body-antrum junction along the lesser curvature, where the surgical repair was performed, and highlights the liver parenchyma (segment II), showing the hepatic penetration site. No active bleeding or biliary injury was observed.

**Figure 4:**
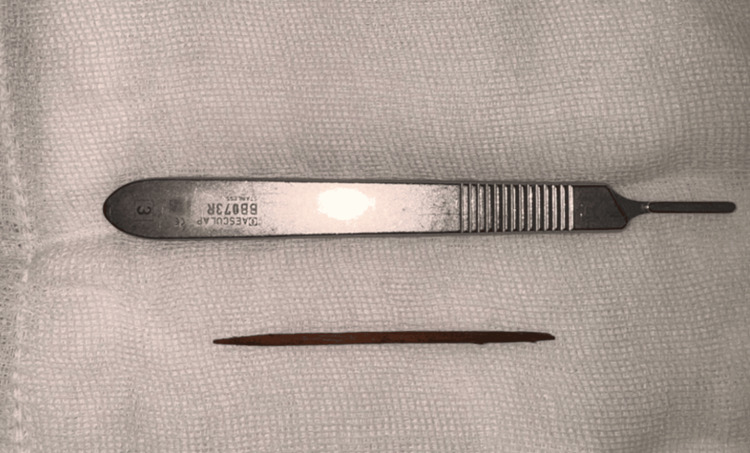
Extracted wooden toothpick positioned below a No. 3 scalpel handle for size reference.

An exploratory laparotomy was performed, revealing a perforation caused by a foreign object (wooden toothpick) (Figure 2) at the anterior wall of the stomach, at the junction of the body and antrum toward the lesser curvature. The object had penetrated segment II of the liver. There was no evidence of free gastrointestinal fluid, hepatic vessel injury, or biliary tree damage. Surgical repair was achieved by primary closure in two layers (Vicryl 2-0), with a continuous suture in the first layer, Lembert stitches in the second layer, and an omental patch (Figures 3, 4).

The patient made a full recovery, tolerating a liquid diet within 24 hours and a soft diet by 48 hours postoperatively. He was discharged home 72 hours after the procedure with no complications. At the time of discharge, the patient was afebrile, tolerating oral intake (soft diet), with no nausea, vomiting, bleeding, or signs of low output. Diuresis, flatus, and bowel movements were present. Follow-up at four weeks showed no long-term complications related to the primary procedure or the laparotomy wound.

## Discussion

The ingestion of foreign bodies is a well-documented clinical scenario, particularly in pediatric and elderly populations. However, ingestion of sharp objects such as wooden toothpicks remains an uncommon but particularly hazardous event due to their potential to cause gastrointestinal perforation, bleeding, sepsis, or death. In a study by Steinbach et al., it was found that perforation occurred in approximately 79% of all toothpick ingestion cases, with a mortality rate close to 10% [5]. Complicating diagnosis, more than half of these patients were unaware of having ingested a toothpick, often leading to delays in intervention.

Our case is consistent with the existing literature; however, it also presents unique features. The patient, a 52-year-old man with a history of chronic alcohol consumption, presented with acute epigastric pain radiating to the back. These nonspecific symptoms, combined with a lack of recall of foreign body ingestion, delayed the diagnosis. Notably, computed tomography failed to detect the toothpick, which aligns with the findings of Erbil et al. and Khan et al., who emphasized the limited sensitivity of radiologic studies in detecting radiolucent foreign objects like wood [6,7]. In this case, exploratory laparotomy revealed a toothpick perforating the anterior wall of the stomach and penetrating segment II of the liver, a rare anatomical trajectory previously reported in only a few cases [5,8].

Toothpicks most commonly perforate the stomach, duodenum, jejunum, and colon. However, migration into solid organs, including the liver, pancreas, or even heart, has also been documented, underscoring the severe consequences of missed diagnoses [7]. In our case, the absence of peritonitis or sepsis, combined with a contained perforation and lack of vascular or biliary injury, allowed for primary repair and rapid recovery. This favorable outcome is uncommon and emphasizes the importance of early surgical intervention.

While endoscopic retrieval is often successful in upper gastrointestinal foreign bodies, with a 98.9% success rate as demonstrated by Yoo et al. [8], surgical management remains the standard in cases involving perforation or suspected migration. European Society of Gastrointestinal Endoscopy (ESGE) guidelines support this approach, recommending urgent endoscopy for sharp-pointed foreign bodies, and immediate surgical consultation when complications are suspected [9,10].

This case highlights the diagnostic challenges and variability in clinical presentation of toothpick ingestion. It reinforces the necessity for clinicians to maintain a high index of suspicion in patients presenting with acute abdominal symptoms and risk factors such as alcohol use, even in the absence of specific ingestion history. Multimodal imaging, although limited for radiolucent materials, and prompt surgical management are crucial to improving patient outcomes.

In conclusion, while rare, toothpick ingestion should remain a differential diagnosis in cases of unexplained abdominal pain. Early recognition and decisive intervention, either endoscopic or surgical, are essential to prevent life-threatening complications. This case contributes to the limited but growing body of evidence surrounding gastric perforation with hepatic involvement, a surgical emergency requiring heightened clinical vigilance.

## Conclusions

Toothpick ingestion is an uncommon but highly dangerous clinical event due to its potential for gastrointestinal perforation and migration into adjacent organs. Diagnosis is frequently delayed given the nonspecific presentation and the radiolucency of wooden materials. This case illustrates a rare but serious complication: gastric perforation with hepatic involvement, successfully managed through timely surgical intervention. Clinicians should maintain a high index of suspicion in patients with unexplained abdominal pain, especially those with risk factors such as chronic alcohol use. Early recognition and appropriate intervention are crucial to preventing severe morbidity or mortality. This report adds valuable information to the limited literature on this condition and supports surgical exploration as the definitive treatment in high-risk scenarios.

## References

[1] Sarici IS, Topuz O, Sevim Y, Sarigoz T, Ertan T, Karabıyık O, Koc A (2017). Endoscopic management of colonic perforation due to ingestion of a wooden toothpick. Am J Case Rep.

[2] Depoorter L, Billiet T, Verhamme M, Moerkercke WV (2019). A toothpick a day, keeps the doctor away?. Acta Gastroenterol Belg.

[3] Ioannidis O, Kakoutis E, Sakkas L, Konstantara A, Chatzopoulos S, Kotronis A, Makrantonakis N (2010). Ingested toothpick fistula of the ileum mimicking Crohn’s disease. Acta Gastroenterol Belg.

[4] Al-Khyatt W, Rashid F, Iftikhar SY (2011). Accidental finding of a toothpick in the porta hepatis during laparoscopic cholecystectomy: a case report. J Med Case Rep.

[5] Steinbach C, Stockmann M, Jara M, Bednarsch J, Lock JF (2014). Accidentally ingested toothpicks causing severe gastrointestinal injury: a practical guideline for diagnosis and therapy based on 136 case reports. World J Surg.

[6] Erbil B, Karaca MA, Aslaner MA, Ibrahimov Z, Kunt MM, Akpinar E, Özmen MM (2013). Emergency admissions due to swallowed foreign bodies in adults. World J Gastroenterol.

[7] Khan S, Ullah S, Anwar M, Athwani R, Nawab K (2023). Bacteremia associated with a toothpick lodged in the duodenal wall. Cureus.

[8] Yoo DR, Im CB, Jun BG (2021). Clinical outcomes of endoscopic removal of foreign bodies from the upper gastrointestinal tract. BMC Gastroenterol.

[9] Birk M, Bauerfeind P, Deprez PH (2016). Removal of foreign bodies in the upper gastrointestinal tract in adults: European Society of Gastrointestinal Endoscopy (ESGE) Clinical Guideline. Endoscopy.

[10] Majjad I, Shubietah AR, Alaqra Y, Alrabi I, AbuMohsen HM, Aburumh H (2023). Perforation of ileum by unnoticed toothpick ingestion presenting as acute appendicitis: a case report. Int J Surg Case Rep.

